# From One Heath to One Sustainability: The Role of Contagious Mastitis Pathogens in Decreasing the Dairy Herd Sustainability

**DOI:** 10.3390/pathogens13100914

**Published:** 2024-10-20

**Authors:** Francesca Zaghen, Valerio M. Sora, Giampaolo Zanirato, Alfonso Zecconi

**Affiliations:** 1 One Health Unit, Department of Biomedical, Surgical and Dental Sciences, School of Medicine, University of Milan, Via Pascal 36, 20133 Milan, Italy; francesca.zaghen@unimi.it (F.Z.); valerio.sora@unimi.it (V.M.S.); 2 Department of Clinical and Community Sciences, School of Medicine, University of Milan, Via Celoria 22, 20133 Milan, Italy; 3 Granlatte Società Cooperativa Agricola, Via Cadriano, 36, 40127 Bologna, Italy; zanirato@granlatte.it

**Keywords:** sustainability, milk, *S. aureus*, risk assessment, milk quality, animal welfare, One Health

## Abstract

Economic, social, and environmental sustainability are the results of efforts aiming to improve all aspects of milk production, respecting animal welfare and improving herd health. An epidemiological study was designed to assess the role of contagious pathogens (*S. aureus* and *S. agalactiae*) in a cohort of 120 dairy herds located in the southern regions of Italy. Milk quality was assessed using certified methods, and the prevalence of mastitis pathogens in bulk tank milk was determined using quantitative polymerase chain reaction. Welfare scores were assessed using a scoring card that has more than 100 items, including animal-based measurements. Statistical analyses were performed using general lineal model and logistic regression procedures. The results showed that *S. aureus* had a significant negative effect on the amount of milk nutrients delivered to the dairy plant, and on the level of welfare, whereas the presence of *S. agalactiae* did not show any significant association. The major risk factors associated with the presence of *S. aureus* were also identified to help prioritize control programs. These results support the “One Sustainability” approach, implying that an increase in animal productivity is related to the improvement of animal health and welfare and potentially leading to the mitigation of greenhouse gas emissions.

## 1. Introduction

Currently, the dairy sector, particularly primary production, is facing new and important challenges that are summarized by the word “sustainability”. This term, in the past, was mainly associated with economic aspects. This is still important, but, currently, sustainability implies environmental and social aspects. In this latter case, animal welfare and the prudent use of antimicrobials, food safety, and security, as well as the supply of nutrients to support the needs of the increasing world population and the demand for high-nutritional-quality foods, are considered. All these different facets of the term “sustainability” are interrelated, and improvements in one area (e.g., economic sustainability) are positively reflected in others, including environmental sustainability [1,2].

Dairy farms have a large impact on environmental health, contributing to greenhouse gas emissions (GHGes) [1]. However, recent studies suggest that high-performing herds can mitigate their environmental impact through better feed conversion efficiency, which reduces GHGe per kg of yield milk, thus improving environmental sustainability [2,3,4]. The main metric used to evaluate the environmental impact of agriculture is usually the GHGe per kg of produced food. This method does not consider the nutritional value of the food, and, inevitably, with this metric, vegetables are less impacting food, but they are also poor in nutrients, especially proteins [5]. Instead, if nutrient density is used as a parameter, animal-based foods have lower GHGe per weight of product than vegetables, whether energy (Kcal), proteins, or total nutrient density is considered [5,6,7,8]. Then, producing more milk with higher quality could help dairy farms to be more efficient, while ensuring healthier and more nutritious food and lessening their environmental impact.

The production of high-quality milk in an efficient and environmentally sustainable manner is closely linked to the respect for animal welfare and the improvement of animal health, resulting in improving the global sustainability of milk production [2,4,6,7,8]. Therefore, the welfare of food-producing animals must become a fully integrated sustainability component [2,9].

In this context, milk quantity and quality are factors that link all of these aspects. Indeed, low quality and/or yield decrease cow welfare, decrease economic and environmental sustainability, and increase the risk for antimicrobial use (AMU), thus decreasing social sustainability. Among the factors affecting milk production, animal health is still the most important, after nutrition [10,11,12], and mastitis still plays a major role [13,14,15]. Reducing the incidence of mastitis in dairy cows is a goal for improving milk quality, safety, security, and sustainability [9]. All of these aspects of mastitis burden should be included in farmers’ decision-making process to improve their herd management, since wrong practices can be the root of the inefficiency in dairy farms and reducing their overall sustainability [8].

Despite the recognized importance of animal health for global herd sustainability, relatively few studies have addressed this topic [1,16,17,18,19,20], and even less have addressed mastitis [9,21,22], in the sustainability scenario. Among mastitis pathogens, contagious ones have the largest impact on milk yield and quality, and they potentially represent a zoonotic risk [23,24,25,26]. The presence of these pathogens is correlated to a decrease in milk yield and quality [27,28], but these specific aspects, to the best of our knowledge, have not yet been investigated from a sustainability perspective, and particularly when GHGes are considered. In particular, the effects on sustainability of the potential reduction in the amount of milk components in weight due to the presence of these infections remain unexplored. These aspects are particularly important for the assessment of a dairy-herd sustainability in countries like Italy, where milk is mainly used for cheese production.

Following the previously described concepts, the Granlatte cooperative (Granlatte Società Cooperativa Agricola, Bologna, Italy), the largest Italian dairy coop, within a broad project aiming to improve the global sustainability of milk production, supported a series of investigations. Within these studies, an epidemiological study was designed to assess the effects of the presence contagious pathogens (*S. aureus* and *S. agalactiae*) on the amount of milk components delivered in a cohort of 120 dairy herds located in the southern regions of Italy (Apulia and Basilicata). This cohort includes all herds that deliver their milk to a national cooperative with a dairy plant in Apulia. This allows continuous and consistent monitoring of milk quality, the same extension service managed by Cooperativa Granlatte (CoG), and very similar environmental and feeding conditions. Specifically, the study aims to quantify the effects of the presence of contagious pathogens on the weight of milk components delivered to CoG to reduce the bias in the GHGe metrics based only on milk yield. Furthermore, the relationship between the level of welfare recorded for each farm and the risk factors associated with the presence of contagious pathogens was also investigated to improve the efficacy of the surveillance and control of these infections in the herd, as a means to mitigate negative effects on the global sustainability of the herd.

## 2. Materials and Methods

### 2.1. Herds and Sampling

The investigation involved 120 Italian dairy farms located in the Apulia and Basilicata regions in South Italy, partners of CoG, and delivering the milk produced to the Cog Apulian plant. All the herd breeds were mainly Italian Holstein cows, together with few Brown Swiss cows.

Bulk tank milk (BTM) samples were collected randomly, biweekly, from each delivery for two years (2021 and 2022).

### 2.2. Milk Quality Assay

After collection, milk samples were immediately stored at 4 °C, delivered to CoG laboratory, and analyzed within 24 h. Milk fat and protein were measured using MilkoScan 7 (Foss, Hilleroed, Denmark)

The amount of milk components delivered was calculated by multiplying the amount of milk delivered (in tons) by the proportion of the single component, and the value obtained (in kg) was considered for statistical analysis.

### 2.3. Contagious Pathogen Assay

Milk samples were analyzed using qPCR with a commercial diagnostic kit (Mastitis 4E kit; DNA Diagnostic A/S, Risskov, Denmark), according to the manufacturer’s instructions. This technique showed sensitivity and specificity, respectively, of ≥0.95 and ≥0.99 for the contagious pathogens [29]. This kit allows for bacterial DNA extraction from, identification of, and quantification of *S. aureus*, *Str. agalactiae*, *M. bovis*, and *Prototheca* spp. These latter two pathogens, *M. bovis* and *Prototheca* spp., were not considered further in this study. Indeed, all samples were negative for *M. bovis*, and *Prototheca* spp. is not a contagious pathogen. A detailed description of the qPCR analytical procedure was previously reported [23]. Each farm was sampled 3 times within 2–3 days, at random, during 2021–2022. The presence of a positive outcome in one of the three consecutive samples was defined as positive for the herd in which the pathogen was recovered.

### 2.4. Welfare Assessment

Welfare assessment (WSA) was based on a scoring card developed specifically for the CoG, covering all the different aspects of dairy animal management. A detailed description of this assessment is out of the scope of this paper, but, in summary, the scoring card was based on 7 major management areas, each containing specific questions, as reported in Table 1. The scoring system mainly includes animal-based measures, which are considered more accurate indicators of welfare [4,30]. The assessment results were then classified into scores, and the sum of scores gives a value that defines the level of welfare of the herd, similar to other approaches applied in Italy (e.g., Classyfarm [31]) and in many other countries. Several questions covered potential risk factors for the presence of contagious pathogens, and they were also considered for further epidemiological analysis.

### 2.5. Statistical Analysis

Data were collected in a database with Excel™ (Microsoft USA, Redmond, DC, USA), and the statistical analysis was performed using the appropriate procedures of SAS 9.4 software (SAS Institute, Cary, NC, USA) and SPSS 29.1 (IBM Corp, Armonk, NY, USA).

Milk-quality data were analyzed by a generalized linear model that applied the GLM procedure of SAS 9.4. The model applied is as follows:

Y_iqgzjk_ = µ + T_i_ + S_q_ + H_g_ + V_z_ + A_j_ + U_k_ + H_g_ × V_z_ + S_q_× H_g_ +A_j_ × (H_g_ + V_z_ + S_q_) + U_k_ × (H_g_ + V_z_ + S_q_) + e_iqgzjk_

where Y = dependent variables (fat, proteins, lactose, and non-fat dry matter); µ = general mean; T_i_ = effect of the year (i = 2021–2022); S_q_ = effect of the season (winter, spring, summer, and autumn); H_g_ = effect of the housing system (deep litter and cubicles); V_z_ = effect of herd size (z = <30; 31–50; 51–80; >80); A_j_ = effect of *Str. agalactiae* results (j = negative, positive); and U_k_ = effect of *S. aureus* results (k = negative, positive).

Welfare assessment scores were analyzed using a simplified GLM model that was equal to the previous model, but without interactions.

The association between the risk factors identified via the WSA and the presence of contagious pathogens in BTM was assessed using a multinomial logistic regression model that included 28 different risk factors, which are detailed in Supplementary Table S1.

## 3. Results

### 3.1. Herd Characteristics

The main characteristics of the 120 dairy farms involved in the study are described in Table 2. In most of the herds, deep litter is used to house animals (63.9%), with a mean size of 48.1 cows/herd, and nearly one-third of the mean herd size (133.2) of the herds housing cows on cubicles (36.1%).

As reported in Table 3, most of the small herds (<50 cows) applied deep-litter housing, while in the larger ones (>50 cows), the cows were mainly in cubicles.

**Figure 1:**
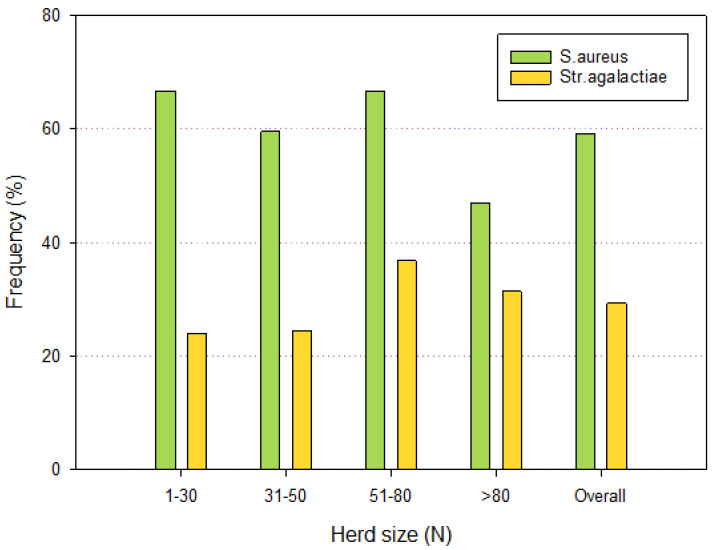
Distribution of positive bulk tank milk by herd size for *S. aureus* and *S. agalactiae* classified.

The analysis of bulk milk for the detection of contagious mastitis pathogens (*S. aureus* and *S. agalactiae*) showed that both pathogens had a higher prevalence in the herds on deep litter when compared to herds with cubicles (Table 4). However, the different distributions among the housing systems were not statistically significant at χ^2^ test (α = 0.05). Figure 1 presents the distribution of positive results for contagious pathogens by herd size. Also in this case, the differences observed were not statistically significant. However, the different distributions among the housing systems were not statistically significant at χ^2^ test (α = 0.05).

### 3.2. Factors Affecting Milk Composition

The significant results of the general linear model statistical analysis are presented in Table 5. Among the factors considered and their interactions, the year, season, and presence of *S. agalactiae* and its interaction with all the other factors, and the interaction between season and housing, were not statistically significant. The resulting models, including statistically significant factors and interactions (Table 5), explained approximately 60% of the parameter variances. Among the different factors, housing and herd size, as expected, showed the most significant values, while the presence of *S. aureus* was very close to the level of α = 0.05. However, the interaction of this latter factor with housing and herd size showed higher levels of significance.

**Figure 2:**
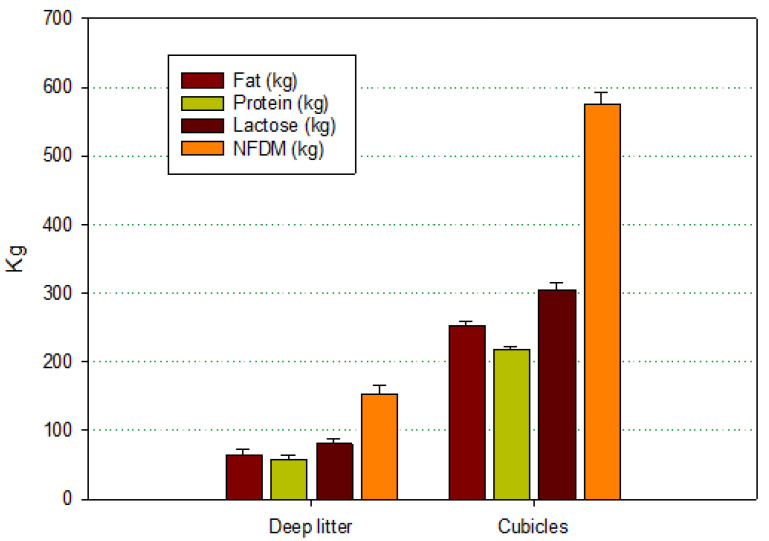
Mean values (±standard deviation) of the amount of milk components delivered on a daily basis, classified by housing systems. All the differences between the two housing systems for all parameters were statistically significant (α = 0.05).

**Figure 3:**
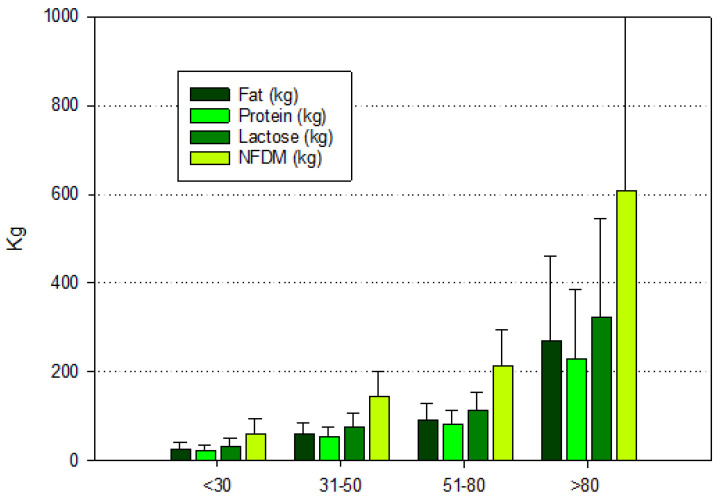
Mean values (±standard deviation) of the amount of milk components delivered on a daily basis, classified by herd size. All differences among herd sizes for all parameters were statistically significant (α = 0.05).

Figure 2 and Figure 3 show the distribution of the quantity of milk components delivered daily to CoG, respectively, by housing and herd size. The overall amount of milk components was higher in the herds with the cubicle housing system. The differences are correlated with the different amounts of milk produced, with a ratio of 2.8 (kg of milk from herds with cubicles/kg of milk from herds with deep litter), but when milk components were considered, this ratio was around 3.8 (kg of nutrients from herds with cubicles/kg of nutrients from herds with deep litter), suggesting that the quality of milk produced by cows in cubicles has a higher nutritional quality.

This latter observation is supported by the mean values observed when the herds were classified by size (Figure 3). The ratio between herds with more than 80 cows and the ones with <30 cows was in the range of 10.1–10.5 for all the parameters, decreased to 4.2–4.3 when herds with 31–50 cows were considered, and then decreased to 2.8–2.9 for herds for 51–80 cows. These data suggest that smaller herds have difficulties obtaining performances close to those of larger herds, probably due to lower efficiency in the management and feeding of the herd.

The differences observed according to housing systems and herd sizes were not unexpected, whereas the analysis of the effects of the presence of *S. aureus* showed interesting results (Table 6). Indeed, the amount of fat, protein, lactose, and non-fat dry matter (NFDM) delivered to the dairy factory was higher in *S. aureus*-negative herds, but the differences were not statistically significant when only deep-litter herds were considered. In contrast, the differences were significant for herds housing cows in cubicles. The differences between each milk component delivered by *S. aureus*-negative herds with cubicles vs. the positive herds were in the range of 18–20%. It may be argued that these differences are biased based on the different herd sizes, but the assessment of the effects of the different herd sizes (Table 7) supports the role of *S. aureus* as a factor that negatively affects the quantity of milk components delivered. Indeed, the analysis of the data classified by herd size and presence of *S. aureus* showed significant differences in milk components among smallest herds (<30 cows) and among the largest (>80 cows). *S. aureus*-negative herds showed higher mean values in the range of 4–10% and in the range of 7–9%, respectively, for smaller and larger herds.

### 3.3. Welfare

The availability of welfare scores allowed us to assess the role of the major factors considered in the GLM analysis on the variance of the WSA scores (Table 8). Only housing and the presence of *S. aureus* in BTM had a significant effect on WSA. The mean value of the herds with cubicles was 19% higher than that of the herds with deep litter, whereas the *S. aureus*-negative herds had a welfare score that was 10% higher than that of the positive herds. The herds were also classified into four categories of welfare (insufficient, sufficient, good, and optimal) by the internal thresholds defined by CoG. Based on this classification, five herds were classified in the optimal class, while only two were classified in the insufficient class, and all the others were in the sufficient class (10) and in the good class (105). The χ^2^ statistical analysis of the distribution of the herd in the four classes according to the housing system was statistically significant (*p* = 0.012). Similarly, the analysis of the effect of the presence of *S. aureus* in BTM showed a significant result (*p* < 0.0001), suggesting that these infections are associated with WSA.

### 3.4. Risk Factors

The availability of information relating to the farm, its structures, its health and milking management, its animal purchases, and the health status of the cows collected through the welfare assessment allowed us to verify the potential associations between these factors and the presence of contagious agents in the farm. Because *S. agalactiae* did not show any significant effects on milk components, we focused on the aspects related to *S. aureus*.

Table 9 summarizes the association between all the considered risk factors and the presence of *S. aureus* infections. Among the 27 risk factors considered, only a few were statistically associated with the presence of *S. aureus* in BTM, as described below.

Only one of the risk factors considered (absence of first stream of milk observation and disposal) was negatively associated (protection) with the presence of *S. aureus* in BTM (odds ratio = 0.04; conf. lim. 95% = 0.002–0.91). This result is certainly unexpected and likely due to a bias related to the small number of herds that did not apply this procedure (5 out of 120). All other statistically significant odds ratios were largely higher than one, suggesting the importance of these factors in increasing the risk of *S. aureus* infections in the herd. To be noticed is that four out of six risk factors are related to milking, and the highest odds ratio was observed for the absence of teat disinfection after milking, supporting the well-known evidence of the role of milking in the spread of intramammary infections [24].

Purchase of animals, as expected, showed a high odds ratio (18.20; 2.98–110.92), since this is one of the common routes of introduction of contagious agents. In fact, in the absence of mandatory checks by the Health Authority and voluntary checks by buyers, the probability of introducing animals (calves, heifers and lactating animals) with *S. aureus* infections is high.

Routine individual milking sampling and analysis (e.g., monthly individual milking sampling operated by Breeder Associations) represents a simple and inexpensive tool for monitoring herd health, as well as the quality of production. Their lack deprives the breeder and his technicians of a practical alarm system, with potential negative consequences, as confirmed by the results of this study, showing a significantly high odds ratio (12.46; 2.70–57.42) for the absence of routine sampling.

## 4. Discussion

The challenges related to the achievement of global sustainability of the dairy herd are different and interrelated. Economic, social, and environmental sustainability are the results of efforts aiming to improve all aspects of milk production. Analogous to the concept of One Health, we can define the process of improving the different areas of sustainability as “One Sustainability”, meaning that a higher level of sustainability in a specific area may be achieved by improving also the other areas of sustainability, and vice versa.

One of the most important challenges in the sustainability assessment is represented by the calculations to define the level of sustainability. A pivotal point in this calculation is the definition of the output. Different methods have been proposed, and most are related to the individual output (cow or herd), represented by the milk produced, in the case of dairy herds [1,12,22]. Usually, this is based on kg of milk; However, in our opinion, this measure may be more accurate if the total amount of milk components is considered, and this is particularly important in areas where most of the milk is used to produce cheese. Moreover, using milk components as a measure of production emphasizes the role of milk as a source of high-quality proteins and nutrients [7,32], thereby making a more accurate comparison with other sources of proteins and nutrients, such as vegetables. Another advantage of using milk-quality data is that they are routinely collected, they are well accepted by farmers, and they are an inexpensive tool for monitoring herds’ health and welfare.

Furthermore, there is increasing evidence of the role of diseases in decreasing not only social and economic sustainability but also environmental sustainability [16,33]. Once more, as for One Health, better herd/cow health will increase the welfare of the animals, reduce the use of AMU, increase efficiency, and mitigate environmental impacts (One Sustainability). In many dairy herds, the most important disease is represented by clinical and subclinical mastitis, particularly contagious mastitis [21,22,23,34].

The results of the statistical analysis of the data collected over two years in 120 herds confirm that few but important factors affect the amount of milk components delivered to the dairy (housing, herd size, and presence of *S. aureus*). None of those may be considered a novelty [35,36,37], but their effects on the amount of milk components were unexpected. Indeed, a decrease in nutrient amounts up to 10% was observed for all of these factors. It is well known that other factors, such as breeds and diets, may influence the milk composition. However, the breeds distribution and the diets are very similar among all the herds considered, overriding these potential effects.

The absence of a significant effect due to the presence of *S. agalactiae* was probably the most unexpected result. We hypothesized that the less pronounced effects of these infections and the proportionally higher frequency in larger herds, when compared with *S. aureus*, biased the overall results. Nevertheless, the absence of a significant effect does not imply that these infections should not be eradicated.

Our study confirmed that hygiene during the milking process and correct milking procedures can greatly influence udder health [38,39,40]. Milking with a bucket had a very high odds ratio (9.16; 1.43–58.61). This result was expected because this type of milking is common in small Italian herds, where management and milking hygiene are frequently poor. Absence or improper post-milking teat disinfection had the highest odds ratio (54.83; 3.11–966.85), confirming the importance of correct teat disinfection after milking as a control factor for the onset of intramammary infections. The presence of a high odds ratio (11.98, 1.32–108.40) when teat disinfection was applied using unregistered products supports the previous observation; moreover, this suggests that the use of unregistered products (in Italy, disinfectant must be authorized by the Ministry of Health based on scientific evidence of efficacy), generally characterized by a low cost but of unknown efficacy, increases the risk of spreading infections, particularly in the case of contagious bacteria.

The high odds ratio associated with the frequent use of oxytocin (10.45; 1.38–78.95) is in agreement with previous observations. Indeed, when the milking procedures are not optimal, milk ejection is impaired [41]. This problem may be exacerbated in the presence of intramammary infections. The use of oxytocin on these farms, therefore, represents the simplest solution to overcome, at least partially, the problem of intramammary infections, but progressively worsens the health status of the udder.

A statistically significant relationship between WSA and herd health was not unexpected [42]. However, to our knowledge, this is the first time that this relationship has been shown when considering the presence of *S. aureus*, and this supports the notion that the control of these infections may have positive outcomes on several aspects of sustainability. Indeed, in addition to increasing milk quality and production, it should also be considered that it would decrease the use of AMU and increase welfare. It should be also noticed that this relationship was detected within herds with an overall good level of welfare.

## 5. Conclusions

There are several critical challenges facing the dairy sector, including being environmentally friendly and, at the same time, increasing food production to cope with the increasing demand and improve economic sustainability, all of which should be performed while maintaining acceptable levels of animal welfare, and food safety and quality. To address these challenges from the health perspective, the One Health approach is considered the best currently available option. However, if we look at these challenges from the production perspective, a “One Sustainability” approach would probably be more effective. This includes pursuing an increase in animal productivity through the improvement of animal health and welfare, potentially leading to the mitigation of GHGe. Indeed, from a global sustainability point of view, the GHGe assessment should consider not only the amount of milk produced (kg) but also the amount of nutrients in order to be comparable with other productions (e.g., vegetables). In addition, it will represent an economic advantage that will improve economic sustainability. From a practical perspective, the results of this study support this approach, showing the negative impact of *S. aureus* on the amount of milk components produced (economic and environmental sustainability) and the relationship between these pathogens with welfare (socials sustainability) and identified the priorities in developing control programs to mitigate these effects.

## Figures and Tables

**Table 1:** Summary of areas of interest and related questions included in the welfare scoring card developed for the Granlatte cooperative.

Area of Interest	Number of Questions/ Observation	Notes
General information	22	
Biosecurity	21	
Lactating cows	22	Including animal-based measures (flank and udder cleanliness, skin lesions, lameness, teat score and cleanliness, and body condition score) and avoidance distance in the barn and at the feeding place.
Nonlactating cows	22	
Heifers	23	
Calves	18	
Milking	20	
Udder	6	Including data on antimicrobial use and the application of preventive measures to decrease AMR.

**Table 2:** Herd sizes for the 120 herds considered, classified by type of housing (cubicles or deep litter).

Housing	N	Mean of Lactating Cows (N)			Mean of Dry Cows (N)			Mean Total Cow (N)		
		Mean	Min	Max	Mean	Min	Max	Mean	Min	Max
**Deep litter**	77 (64.2%)	40.3	5	113	7.8	0	33	48.1	8	140
**Cubicles**	43 (35.8%)	111.3	12	460	21.9	2	125	133.2	14	585
**Total**	120	65.9	5	460	12.9	0	125	78.8	8	585

**Table 3:** Distribution of housing systems by herd size among the 120 herds considered.

Size	Cubicles	Deep Litter	Total
1–30	2 (4.7%) ^a,1^	19 (24.7%) ^b^	21 (17.5%)
31–50	6 (14.0%) ^a^	31 (40.3%) ^b^	37 (30.8%)
51–80	13 (30.2%) ^a^	17 (22.1%) ^a^	30 (25.0%)
>81	22 (51.2%) ^a^	10 (13.0%) ^b^	32 (26.7%)
Total	43 (35.8%)	77 (64.2%)	120 (100%)

^1^ Different superscript letters within the same herd size represent a statistically different proportion (*p* < 0.05).

**Table 4:** Distribution of positive molecular analysis results for the detection of *S. aureus* and *S. agalactiae* in the 120 herds by type of housing.

Housing	N	*S. aureus*			*S. agalactiae*		
		Frequency (%)	Lower 95% Limit	Upper 95% Limit	Frequency (%)	Lower 95% Limit	Upper 95% Limit
**Deep litter**	77	64.93	57.40	72.47	33.77	26.30	41.23
**Cubicles**	43	48.83	38.27	59.40	20.93	12.33	29.53
**Total**	120	59.16	52.95	65.38	29.16	23.41	34.92

**Table 5:** Statistically significant factors estimated by GLM statistical analyses affecting the quantity of milk and milk components delivered by the 120 herds considered.

Parameter		Factors					R^2^
	Housing	Herd Size	Positivity to *S. aureus*	Housing × Size	Housing × Positivity to *S. aureus*	Herd Size × Positivity to *S. aureus*	
**Fat (kg)**	<0.0001	<0.0001	0.0391	<0.0001	0.0180	0.0064	59.9%
**Proteins (kg)**	<0.0001	<0.0001	0.0503	<0.0001	0.0119	0.0042	61.2%
**Lactose (kg)**	<0.0001	<0.0001	0.0520	<0.0001	0.0158	0.0077	60.0%
**NFDM ^1^ (kg)**	<0.0001	<0.0001	0.0530	<0.0001	0.0146	0.0078	60.3%
**Milk delivered (ton)**	<0.0001	<0.0001	0.0541	<0.0001	0.0155	0.0109	59.8%

^1^ Non-fat dry matter.

**Table 6:** Mean amount of milk components classified by housing system and bulk tank milk positivity for *S. aureus* (mean ± std. dev.).

Housing	Status	Fat	Protein	Lactose	NFDM ^1^
**Deep litter**	*S. aureus*	61.76 ± 40.82 ^a,2^	54.26 ± 35.94 ^a^	76.18 ± 49.82 ^a^	144.12 ± 94.23 ^a^
	Negative	70.36 ± 42.72 ^a^	62.17 ± 36.21 ^a^	86.81 ± 49.28 ^a^	164.27 ± 93.91 ^a^
**Cubicles**	*S. aureus*	196.19 ± 190.69 ^a^	168.75 ± 157.73 ^a^	236.21 ± 224.86 ^a^	445.79 ± 421.82 ^a^
	Negative	235.55 ± 180.42 ^b^	200.31 ± 150.20 ^b^	279.76 ± 214.21 ^b^	528.88 ± 402.52 ^b^

^1^ Non-fat dry matter. ^2^ Different superscripts within the same housing system indicate statistically different difference (*p* < 0.05).

**Table 7:** Mean amount of milk components classified by herd size and bulk tank milk positivity for *S. aureus* (mean ± standard deviation).

Size	Status	Fat	Protein	Lactose	NFDM ^1^
**1–30**	*S. aureus*	26.51 ± 12.31 ^a,2^	22.67 ± 11.04 ^a^	32.03 ± 15.04 ^a^	60.61 ± 28.66 ^a^
	Negative	27.25 ± 14.50 ^b^	24.32 ± 13.30 ^b^	35.41 ± 19.00 ^b^	66.02 ± 35.43 ^b^
**31–50**	*S. aureus*	57.22 ± 21.37 ^a^	50.06 ± 18.68 ^a^	70.07 ± 25.83 ^a^	132.64 ± 48.78 ^a^
	Negative	69.72 ± 25.40 ^a^	62.21 ± 22.67 ^a^	87.06 ± 31.48 ^a^	164.45 ± 59.20 ^a^
**51–80**	*S. aureus*	86.11 ± 35.16 ^a^	76.02 ± 31.05 ^a^	106.44 ± 41.30 ^a^	201.15 ± 78.66 ^a^
	Negative	106.17 ± 33.26 ^a^	93.00 ± 29.39 ^a^	127.54 ± 36.66 ^a^	242.63 ± 71.43 ^a^
**>81**	*S. aureus*	257.05 ± 198.01 ^a^	221.20 ± 161.66 ^a^	310.03 ± 231.72 ^a^	585.19 ± 433.73 ^a^
	Negative	281.38 ± 181.53 ^b^	238.35 ± 150.84 ^b^	333.49 ± 216.10 ^b^	630.33 ± 405.39 ^b^

^1^ Non-fat dry matter. ^2^ Different superscripts within the same herd size indicate statistically different differences (*p* < 0.05).

**Table 8:** Significant results of the general linear model analysis on the effects of the main factors on welfare score variance (model R^2^ = 0.17).

	Housing ^1^		*S. aureus* ^1^	
	Deep Litter	Cubicles	Positive	Negative
Mean	20.140	23.920	20.625	227.543
Standard deviation	55.84	42.16	45.40	55.51

^1^ Means within the two categories of each factor (housing, *S. aureus*) are statistically different at α = 0.05.

**Table 9:** Significant risk factors identified using multinomial logistic regression model with the presence of *S. aureus* as response (disease) variables and 27 risk factors.

Risk Factors	Odds Ratio	95% Confidence Interval		*p*
		Inferior Limit	Superior Limit	
Bucket milking vs. parlor milking	9.16	1.43	58.61	0.019
Absence of forestripping milk observation and disposal vs. presence	0.04	0.002	0.91	0.043
Absence or unproper post-milking teat disinfection vs. proper disinfection	54.83	3.11	966.85	0.006
Post-milking teat disinfection with a non-authorized product vs. proper disinfection	11.98	1.32	108.40	0.027
Frequent use of oxytocin vs. no use	10.45	1.38	78.95	0.023
Animal purchase vs. no purchase	18.20	2.98	110.92	0.002
Absence of monthly individual milk analysis vs. presence	12.46	2.70	57.42	0.001

## Data Availability

Data are unavailable due to privacy or ethical restrictions included in the contract between farmers and Granlatte Società Cooperativa Agricola.

## Supplementary Materials

The following supporting information can be downloaded at https://www.mdpi.com/article/10.3390/pathogens13100914/s1, Supplementary Table S1: Logistic regression analysis on the 28 risk factors considered.

## References

[1] Capper J.L., Cady R.A. (2020). The effects of improved performance in the US dairy cattle industry on environmental impacts between 2007 and 2017. J. Anim. Sci..

[2] Buller H., Blokhuis H., Jensen P., Keeling L. (2018). Towards Farm Animal Welfare and Sustainability. Animals.

[3] Froldi F., Lamastra L., Trevisan M., Mambretti D., Moschini M. (2022). Environmental impacts of cow’s milk in Northern Italy: Effects of farming performance. J. Clean. Prod..

[4] Kristensen T., Mogensen L., Knudsen M.T., Hermansen J.E. (2011). Effect of production system and farming strategy on greenhouse gas emissions from commercial dairy farms in a life cycle approach. Livest. Sci..

[5] Pasinato S., Ferrero F., Rolando G., Comino L., Tabacco E., Borreani G. (2023). A Living Lab approach for sustainable intensification of dairy production: A case study of an organic and a conventional farm in northern Italy. Eur. J. Agron..

[6] Capper J.L. (2023). The impact of controlling diseases of significant global importance on greenhouse gas emissions from livestock production. One Health Outlook.

[7] Drewnowski A. (2018). Measures and metrics of sustainable diets with a focus on milk, yogurt, and dairy products. Nutr. Rev..

[8] Werner L.B., Flysjö A., Tholstrup T. (2014). Greenhouse gas emissions of realistic dietary choices in Denmark: The carbon footprint and nutritional value of dairy products. Food Nutr. Res..

[9] Vieux F., Soler L.G., Touazi D., Darmon N. (2013). High nutritional quality is not associated with low greenhouse gas emissions in self-selected diets of French adults. Am. J. Clin. Nutr..

[10] Hultgren J., Hiron M., Glimskar A., Bokkers E.A.M., Keeling L.J. (2022). Environmental Quality and Compliance with Animal Welfare Legislation at Swedish Cattle and Sheep Farms. Sustainability.

[11] Gülzari S., Ahmadi B.V., Stott A.W. (2018). Impact of subclinical mastitis on greenhouse gas emissions intensity and profitability of dairy cows in Norway. Prev. Vet. Med..

[12] Villettaz Robichaud M., Rushen J., de Passillé A.M., Vasseur E., Haley D., Pellerin D. (2019). Associations between on-farm cow welfare indicators and productivity and profitability on Canadian dairies: II. On tiestall farms. J. Dairy Sci..

[13] Villettaz Robichaud M., Rushen J., de Passillé A.M., Vasseur E., Orsel K., Pellerin D. (2019). Associations between on-farm animal welfare indicators and productivity and profitability on Canadian dairies: I. On freestall farms. J. Dairy Sci..

[14] Place S.E., Mitloehner F.M. (2010). Invited review: Contemporary environmental issues: A review of the dairy industry’s role in climate change and air quality and the potential of mitigation through improved production efficiency. J. Dairy Sci..

[15] Puerto M.A., Shepley E., Cue R.I., Warner D., Dubuc J., Vasseur E. (2021). The hidden cost of disease: I. Impact of the first incidence of mastitis on production and economic indicators of primiparous dairy cows. J. Dairy Sci..

[16] Capper J.L., Williams P. (2023). Investing in health to improve the sustainability of cattle production in the United Kingdom: A narrative review. Vet. J..

[17] Mostert P.F., van Middelaar C.E., de Boer I.J.M., Bokkers E.A.M. (2018). The impact of foot lesions in dairy cows on greenhouse gas emissions of milk production. Agric. Syst..

[18] ADAS (2015). Study to Model the Impact of Controlling Endemic Cattle Diseases and Conditions on National Cattle Productivity, Agricultural Performance and Greenhouse Gas Emissions. Final Report to Defra/AHVLA on Project FFG1016. (No. AC0120).

[19] Hospido A., Sonesson U. (2005). The environmental impact of mastitis: A case study of dairy herds. Sci. Total Environ..

[20] Mostert P.F., Bokkers E.A.M., de Boer I.J.M., van Middelaar C.E. (2019). Estimating the impact of clinical mastitis in dairy cows on greenhouse gas emissions using a dynamic stochastic simulation model: A case study. Animal.

[21] Sora V.M., Panseri S., Nobile M., Di Cesare F., Meroni G., Chiesa L.M., Zecconi A. (2022). Milk Quality and Safety in a One Health Perspective: Results of a Prevalence Study on Dairy Herds in Lombardy (Italy). Life.

[22] Zecconi A. (2007). Contagious mastitis control. FIL-IDF Bull..

[23] Zaghen F., Sora V.M., Meroni G., Laterza G., Martino P.A., Soggiu A., Bonizzi L., Zecconi A. (2023). Epidemiology of Antimicrobial Resistance Genes in *Staphylococcus aureus* Isolates from a Public Database from a One Health Perspective—Sample Origin and Geographical Distribution of Isolates. Antibiotics.

[24] Zaghen F., Sora V.M., Meroni G., Laterza G., Martino P.A., Soggiu A., Bonizzi L., Zecconi A. (2023). Epidemiology of Antimicrobial Resistance Genes in *Staphyloccocus aureus* Isolates from a Public Database in a One Health Perspective—Sample Characteristics and Isolates&rsquo; Sources. Antibiotics.

[25] Meroni G., Sora V.M., Martino P.A., Sbernini A., Laterza G., Zaghen F., Soggiu A., Zecconi A. (2022). Epidemiology of Antimicrobial Resistance Genes in *Streptococcus agalactiae* Sequences from a Public Database in a One Health Perspective. Antibiotics.

[26] Crestani C., Forde T.L., Lycett S.J., Holmes M.A., Fasth C., Persson-Waller K., Zadoks R.N. (2021). The fall and rise of group B *Streptococcus* in dairy cattle: Reintroduction due to human- to- cattle host jumps?. Microb. Genom..

[27] Haag A.F., Fitzgerald J.R., Penadés J.R. (2019). *Staphylococcus aureus* in Animals. Microbiol. Spectr..

[28] Zecconi A., Calvinho L.F., Fox K.L. (2006). *Staphylococcus aureus* intramammary infections. IDF Bull..

[29] Paradis M.E., Haine D., Gillespie B., Oliver S.P., Messier S., Comeau J., Scholl D.T. (2012). Bayesian estimation of the diagnostic accuracy of a multiplex real-time PCR assay and bacteriological culture for 4 common bovine intramammary pathogens. J. Dairy Sci..

[30] EFSA Panel on Animal Health and Welfare (AHAW) (2012). Statement on the use of animal-based measures to assess the welfare of animals. EFSA J..

[31] Ventura G., Lorenzi V., Mazza F., Clemente G.A., Iacomino C., Bertocchi L., Fusi F. (2021). Best Farming Practices for the Welfare of Dairy Cows, Heifers and Calves. Animals.

[32] Baber J.R., Sawyer J.E., Wickersham T.A. (2018). Estimation of human-edible protein conversion efficiency, net protein contribution, and enteric methane production from beef production in the United States. Transl. Anim. Sci..

[33] Chetroiu R., Cismileanu A.E., Cofas E., Petre I.L., Rodino S., Dragomir V., Marin A., Turek-Rahoveanu P.A. (2022). Assessment of the Relations for Determining the Profitability of Dairy Farms, A Premise of Their Economic Sustainability. Sustainability.

[34] Zecconi A., Dell’Orco F., Rizzi N., Vairani D., Cipolla M., Pozzi P., Zanini L. (2019). Cross-sectional study on the prevalence of contagious pathogens in bulk tank milk and their effects on somatic cell counts and milk yield. Ital. J. Anim. Sci..

[35] Dufour S., Fréchette A., Barkema H.W., Mussell A., Scholl D.T. (2011). Invited review: Effect of udder health management practices on herd somatic cell count. J. Dairy Sci..

[36] Robles I., Kelton D.F., Barkema H.W., Keefe G.P., Roy J.P., von Keyserlingk M.A.G., DeVries T.J. (2020). Bacterial concentrations in bedding and their association with dairy cow hygiene and milk quality. Animal.

[37] Henriksson M., Flysjö A., Cederberg C., Swensson C. (2011). Variation in carbon footprint of milk due to management differences between Swedish dairy farms. Animal.

[38] Silk A.S., Fox L.K., Hancock D.D. (2003). Removal of Hair Surrounding the Teat and Associated Bacterial Counts on Teat Skin Surface, in Milk, and Intramammary Infections. J. Vet. Med. Ser. B.

[39] Zecconi A., Piccinini R., Fox K.L. (2003). Epidemiologic study of intramammary infections with *Staphylococcus aureus* during a control program in nine commercial dairy herds. JAVMA.

[40] Mein G.A. (2012). The Role of the Milking Machine in Mastitis Control. Vet. Clin. N. Am. Food Anim. Pract..

[41] Zecconi A., Frosi S., Cipolla M., Gusmara C. (2018). Effects of chronic mastitis and its treatment with ketoprofen on the milk ejection curve. J. Dairy Res..

[42] Authority E.F.S. (2009). Scientific opinion on welfare of dairy cows in relation to udder problems based on a risk assessment with special reference to the impact of housing, feeding, management and genetic selection. EFSA J..

