# Supplementary material for: From One Heath to One Sustainability: The Role of Contagious Mastitis Pathogens in Decreasing the Dairy Herd Sustainability
Source: Pathogens. 2024 Oct 20;13(10):914. doi: 10.3390/pathogens13100914 (PMC11510415; doi:10.3390/pathogens13100914)
Supplement: Supplementary file 1 [file pathogens-13-00914-s001.zip › pathogens-3219371-supplementary.pdf]

Supplementary table 1: Logistic regression analysis on the 28 risk factors considered

Factors	B	Standard error	Sign.	Exp(B)	95% confidence interval for Exp(B)	
					Lower limit	Upper limit
Intercept	-4,005	1,725	0.020			
Number of cows	,008	,006	,174	1,008	,996	1,020
N. of milking operators	,073	,676	,913	1,076	,286	4,048
Altered teat proportion (%)	,121	,070	,083	1,129	,984	1,295
Proportion of animals with clean udders (%)	-,008	,013	,380	,537	,992	,967
Deep litter (vs. cubicles)	-1,385	,815	,089	,250	,051	1,238
No bacteriological analysis on bulk tank milk (vs. presence)	-,990	1,714	,564	,372	,013	10,697
No therapy at dry-off (vs. selective dry cow therapy with protocol)	-,085	1,182	,943	,918	,091	9,313
Selective dry cow therapy without protocol (vs. Selective dry cow therapy with protocol)	,102	,932	,913	1,107	,178	6,878
Use of not registered products for therapy (vs. registered)	1,100	1,066	,302	3,004	,372	24,282
Use of not registered and registered products for therapy (vs. registered)	,681	1,837	,711	1,976	,054	72,337
No use of teat sealant (vs. use)	,222	,863	,797	1,249	,230	6,781
Bucket or milk-pipeline in free-stall (vs. milk parlour)	2,215	,947	,019	9,161	1,432	58,611
No teat cleansing (vs. cleansing)	-1,946	,938	,038	,143	,023	,898
Teat cleansing with water (vs. cleansing foam)	-1,406	,958	,142	,245	,038	1,602
Incorrect teat drying (vs. correct)	,508	1,327	,702	1,662	,123	22,393
No teat drying (vs. correct)	-,618	1,167	,596	,539	,055	5,308
Occasional or absent forestripping and observation of the first milk streams (vs. forestripping and observation)	-3,251	1,609	,043	,039	,002	,907
Insufficient or absent teat disinfection (vs. disinfection with registered products)	4,004	1,464	,006	54,830	3,109	966,855
Teat disinfection with unregistered products (vs. disinfection with registered products)	2,483	1,124	,027	11,983	1,325	108,405

Frequent oxytocin use (vs. no use)	2,347	1,032	,023	10,453	1,384	78,952
Occasional oxytocin use (vs. no use)	,858	1,016	,398	2,357	,322	17,256
Calving pen: absence or calving in cubicles (vs. presence)	1,140	0,692	0,100	0,3127	0,805	12,147
Animals purchase: from 1 to 5 animals (vs.0)	2,902	,922	,002	18,202	2,987	110,924
Absence of routine monthly milk sampling and analysy (vs. presence)	2,523	,779	,001	12,464	2,705	57,420
No nursing (vs. presence)	1,073	,702	,126	2,924	,739	11,567
No holding area (vs. presence)	-,951	,631	,132	,386	,112	1,332
No hospital pen (vs. presence)	-1,092	,933	,242	,336	,054	2,088
